# MULTIDISCIPLINARY EMERGING PERSPECTIVES ON BUILDING AND MAINTAINING NETWORKS IN AGING

**DOI:** 10.1093/geroni/igz038.2070

**Published:** 2019-11-08

**Authors:** Eugenie Stephenson

**Affiliations:** University of Maryland Baltimore County, Baltimore, Maryland, United States

## Abstract

This presentation is a reflective piece on developing and staging of the inaugural Gerontology Student Workforce Day at the Georgia Capitol held in January 2016 through coordination with the state-level Council on Aging (GCOA: Georgia-Council-on-Aging). The aims of this initiative focused on bridging students’ gerontology education and career aims with current legislative concerns for older adults at the state level through networking and advocacy efforts. We also sought out to highlight to state legislators the necessity to support gerontology education. Results of this networking engagement included educating state legislators on both the role of gerontology education to support the needs of older residents at the community-level and highlighting to both parties the impact of gerontology professionals on the state’s workforce. As a result, we engaged gerontology students and early career aging professionals in high-impact networking opportunities focused on service and policy efforts with state legislators and local AAAs (area-agencies-on-aging).

